# Treatment persistence and adherence and their consequences on patient outcomes of generic versus brand-name statins routinely used to treat high cholesterol levels in Spain: a retrospective cost-consequences analysis

**DOI:** 10.1186/s12944-018-0918-y

**Published:** 2018-12-06

**Authors:** A. Sicras-Mainar, L. Sánchez-Álvarez, R. Navarro-Artieda, J. Darbà

**Affiliations:** 1 Scientific Directorate, ClinicResearch, Rovira i Virgili, 10, 08391 Tiana, Barcelona, Spain; 2 Primary Care Pharmacy Directorate, Avilés, Asturias Spain; 3 Department of Medical Documentation, Hospital Germans Trias i Pujol, Badalona, Barcelona, Spain; 4 Department of Economics, Universitat de Barcelona, Barcelona, Spain

**Keywords:** Hypercholesterolemia, Adherence, Persistence, Costs, Outcomes, patient’s at goal, Cardiovascular events, Generics, Brand-name

## Abstract

**Background:**

High blood lipoprotein concentrations are one of the major risk factors for cardiovascular diseases. Drug therapy is the base of treatment; statins in particular. Both brand-name and generic presentations are available for statin therapy of high cholesterol levels. Factors that may influence their use in routine medical practice include, among others, patient persistence and adherence to treatment as prescribed by physicians. The aim of this retrospective analysis was to provide real-world evidence of treatment persistence and adherence and their consequences on economic and patient outcomes of generic versus brand-name statins routinely used to treat high cholesterol levels in Spain.

**Methods:**

Existing real-world electronic medical records abstracted from a database of two regions in Spain were analyzed. The analysis compared generic versus brand-name statins data from subjects’ who started treatment between July 1, 2010 and June 30, 2012. Treatment persistence, adherence expressed as medication possession ratio (MPR), healthcare resource utilization and their costs were analyzed together with patient’s at-goal rates of low-density-lipoprotein-cholesterol (LDL-c), incidence of any major cardiovascular event (CVE) and all-cause mortality during a 5-year follow-up period. Multivariate analyses were applied.

**Results:**

A total of 13,244 records were included. Persistence was lower with generics; adjusted hazard ratio -HR- [95% confidence interval]: 0.86 [0.82–0.91], *p* < 0.001) and MPR was also lower: 61.5% vs. 65.1% (*p* < 0.001). Less patients with generics reached their LDL-c goal: 39.2% [38.3–40.2%] vs. 42.0% [40.2–43.7%]; adjusted odds ratio; 0.87 [0.80–0.95], *p* = 0.003. Compared to brand-name statins, the observed probability of occurrence of a CVE; HR: 1.31 [1.15–1.50], *p* < 0.001, and also all-cause deaths; HR: 1.36 [1.15–1.62], was significantly higher with generics; *p* < 0.001 in both cases. Adjusted mean total healthcare cost per patient was also higher with generic than with brand-name statins: €9118 (9059–9176) vs. €7980 (7853–8808) [adjusted difference: €1137 (997–1277), *p* < 0.001].

**Conclusion:**

This retrospective cost-consequences analysis found poorer treatment persistence and adherence in patients who first started therapy with generic instead of brand-name statins in routine medical practice in Spain. Also, patients receiving generics were more unlikely to reach LDL-c goals, showed increased probability of having CVE and all-cause mortality at a higher cost to payers.

**Electronic supplementary material:**

The online version of this article (10.1186/s12944-018-0918-y) contains supplementary material, which is available to authorized users.

## Background

Cardiovascular disease (CVD) is one of the leading causes of morbidity and mortality in industrialized countries, despite the advances observed in clinical outcomes [1, 2]. Preventing and managing CVD in these patients is a priority objective of healthcare systems [2, 3]. The importance of prevention is therefore unquestionable, and must be applied at different levels: a) in the general population, by promoting healthy lifestyle habits, and b) individually, for individuals with moderate/high risk of CVD or established CVD, and reducing high cardiovascular risk factors such as hypertension and dyslipidemia [3–5]. Dyslipidemias are a set of asymptomatic diseases caused by abnormal blood lipoprotein concentrations, and are one of the major risk factors for cardiovascular diseases in adults [5]. Drug therapy forms one of the bases of treatment; lipid-lowering agents in general, and statins in particular (simvastatin, atorvastatin, etc.), are drugs that reduce cholesterol synthesis in the liver by a mechanism of competitive inhibition with HMG-CoA reductase [6]. In spite of evidence supporting the efficacy of statins in the prevention of major cardiovascular events, lack of treatment adherence continues to be a considerable problem [7]. Several studies have shown that lack of adherence to statin treatment remains above 50% [6–8]. Thus, long-term therapeutic non-compliance is one of the major problems in daily practice, as it results in lower clinical effectiveness, lack of achievement of treatment goals and a possible increase in the use of healthcare resources [9]. Some factors associated with lack of adherence to statin treatment have been identified, such as: their use in primary prevention, low economic income, young age, poly-medication, absence of symptoms, psychological comorbidities, adverse reactions and/or unhealthy lifestyle habits [9, 10].

At present, both brand-name and generic presentations are available for statin therapy of high blood cholesterol levels. Generics are drugs that are bioequivalent to the original brand name and have the same levels of efficacy, safety and quality [11]. Factors that may influence their use in routine medical practice include physician awareness and local or national healthcare intervention strategies with respect to generic drugs [12]. It should be noted that current policies in Spain regarding the price restriction of brand-name versus generic drugs no longer constitute such a solid argument as to demand their use at the same price (reference prices require price equality that can be financed by the Spanish National Health System –NHS-) [13].Discrepancies are seen between arguments in favor of and against prescribing generic drugs [14, 15]. The disadvantage of a generic drug is the confusion it may cause for patients regarding its commercial name (active substance) and its presentation or form (bio-appearance), especially in older people [14, 16, 17]. This potential confusion may lead to medication errors, which could in turn lead to treatment non-adherence, cause a possible decrease in clinical effectiveness, trigger the onset of adverse effects and generate a potential increase in associated healthcare costs [18–20]. The change in pill appearance (physical characteristics of shape, color, size and packaging that identify medicines) which occurs when generic drugs are supplied by different brands over time might result in higher levels of treatment discontinuation or mean that patients are less likely to adhere to treatment [19]. Therefore, administration of a generic drug could be considered a factor to be taken into account, particularly in some countries, like Spain, where generic substitution is allowed and their governments encourage doctors to prescribe them. Pharmacists can make a substitution between generics, choosing one from those available in the national reference pricing system, unless the doctor and/or the patient prefer another product [19]. Treatment persistence constitutes a key factor in disease progression and risk of complications [20]. Confirming this hypothesis (link between treatment persistence vs. clinical and economic consequences) with the same active substance would render the conclusions more robust.

While there are other data sources, including claims databases, patient registries, internet-based consumer research and prescription-based data collection, this article focuses on the evidence with respect to behaviors in Spain. This allows for an established method of researching current treatment practices across a wide range of disease areas using robust, real-world data that accurately reflect current symptom prevalence and severity as well as associated treatment practices for a number of common chronic disease areas. This article provides real-world evidence on treatment persistence and medication possession as well as clinical (patients reaching goals and incidence of a cardiovascular event) and economic consequences of generic versus brand-name drugs used in routine clinical practice to treat high blood cholesterol levels with statins.

## Methods

### Design

In order to provide real-world evidence of treatment persistence and economic and patient outcomes of generic versus brand-name statin drugs routinely used to treat high cholesterol levels in Spain, we hypothesized that using generics would be associated with lower persistence and a lower medication possession ratio (worse drug adherence) than their counterpart brand-name statins. This could be due to the impact of different bio-appearances of generics among them and versus brand-name statins. To answer such hypotheses, this paper reported the findings of a secondary investigational analysis conducted by ClinicResearch that was submitted and approved by the Institutional Research Board of the Universitat Internacional de Catalunya in Barcelona and the Spanish Agency of Medicines. This research used existing anonymized electronic medical records (EMRs) linked to the patient database of the RedISS Foundation (http://rediss.es/). The RedISS Foundation is a research network whose primary goals are to carry out research on the services provided by healthcare management organizations in Spain. RedISS is a longitudinal, anonymized database of EMRs kept by primary healthcare physicians across Spain. The patient data included in the database are stripped of identifying details as specified in Spanish Law 15/1999, of 13 December, on Personal Data Protection. For the findings presented here, primary healthcare centers in two regions (Catalonia and Asturias) provided patient data in the form of clinical records for over 343,182 actively registered individuals. These two regions were selected because of the availability of data to cover the longitudinal period of the research. The data are representative of the Spanish population. The data available include information on demographics, medical history (including diagnoses and health contacts), results of clinical research, drug prescriptions and days of sick leave. Diagnostic data are recorded using the International Classification of Primary Care version 2 (ICPC-2) and/or the International Statistical Classification of Diseases and Related Health Problems (Ninth Revision) codes [21].

### Patient and public involvement

Patients or the public were not involved directly in this work, but EMRs were abstracted from the database to carry out the analysis. Records with a diagnosis of hypercholesterolemia were identified in the database based on patients’ medical and treatment history. Patients first prescribed atorvastatin or simvastatin (brand-name or generic) between July 1, 2010, and June 30, 2012, were eligible to enroll. The inclusion criteria were as follows: male or female, 18 years of age or older, having been entered in the database 12 or more months before first being prescribed atorvastatin or simvastatin, having been enrolled in the long-term prescription follow-up program at each healthcare center, having received ≥2 prescriptions for generic or brand-name statins and having been diagnosed with hypercholesterolemia before the date of enrollment, with at least 2 follow-up contacts in the database. Patients first prescribed atorvastatin or simvastatin after June 30, 2012, and patients who might have been exposed to these statins within 12 months of the index date, were excluded. Patients who received combination therapy with concomitant or sequential generic or brand-name statins were considered ineligible for the analysis, as were those receiving a statin different from the two evaluated here. The EMRs of patients whose healthcare was transferred out to other regions or healthcare centers during the follow-up period were also excluded. From these data, four subgroups of therapeutic regimens were identified according to the Anatomical Therapeutic Chemical (ATC) classification system [22]: brand-name atorvastatin, generic atorvastatin, brand-name simvastatin and generic simvastatin (ATC, atorvastatin: C10AA05; simvastatin: C10AA01). The index date was defined as the date on which a patient was first prescribed either a brand-name or generic statin. Patients were followed up until the earliest date among the following options: the index date plus 60 months; the completion of recorded data; the last prescription for the regimen of interest plus 30 days; or the date of regimen change. The recorded data completion date was defined as the earliest date from among the following: the last date on which data was collected for the practice, the incidence of a cardiovascular event or the date of all-cause mortality. The initial analysis plan included obtaining all available records that met all the screening criteria in the enrollment period (from the index date). Therefore, no initial predetermination of the minimum sample size was performed. EMRs fulfilling inclusion and exclusion criteria as mentioned above were abstracted in one shot and transferred to a database in Microsoft Access software, then, transferred to a statistical package (IBMSPSS v20) for analysis. The abstraction was performed by means of an algorithm which included all variables described in the following sections of methods.

### Diagnosis and demographics

The records of patients with hypercholesterolemia were obtained from the International Classification of Primary Care (ICPC-2; T93) [21] and/or the International Classification of Diseases (Ninth Revision), Clinical Modification (ICD-9-CM; 272.0). The diagnosis of hypercholesterolemia was always at the physician’s discretion, following scientific society recommendations (2016 European Society of Cardiology/European Atherosclerosis Society Guidelines for the Management of Dyslipidaemias) [3]. The sociodemographic and comorbidity variables were as follows: age (continuous and by range) and sex, as well as personal history based on the ICPC-2 of hypertension (K86, K87), diabetes mellitus (T89, T90), obesity (T82), active smoking (P17), alcohol abuse (P15, P16), all types of organ failure (heart, liver and kidney), ischemic heart disease (codes: K74, K76, K75), cerebrovascular accident (K90, K91, K93), depressive syndrome (P76) and malignant neoplasms (all types: A79, B72–75, D74–78, F75, H75, K72, L71, L97, N74–76, T71–73, U75–79, W72–73, X75–81, Y77–79). As a summary variable of the general comorbidity, for each patient treated, the following were used: a) the Charlson comorbidity index [23], as an approximation to the severity of the patient, b) the number of chronic comorbidities and c) the case-mix index, based on adjusted clinical groups, a system for classifying patients according to similar resource consumption [24].

### Treatments

Two groups were distinguished based on the initial treatment: a) brand-name statins, and b) generic statins, with no combinations. The follow-up period was 5 years, starting from the date of patient inclusion. The index date was the treatment (atorvastatin or simvastatin) start date, while the end date was whichever of the following occurred first: a) end of follow up date (5 years follow-up), b) occurrence of a cardiovascular event/death, c) switch to another lipid-lowering treatment other than the one that led to the patient’s inclusion, and d) cessation/abandonment of the medication. The information was obtained from the drug supply records for drugs. The choice of brand-name or generic drug for a specific patient was at the physician’s discretion (*routine clinical practice*). To fulfill the initial analysis plan according to the objective of this research, records of simvastatin and atorvastatin were separated into two groups depending on whether they included a brand-name or a generic statin (see Additional file 1: Tables S1 and S2 and Table 2). Patients first prescribed atorvastatin or simvastatin (brand-name or generic) between July 1, 2010, and June 30, 2012, were eligible to enroll. Only atorvastatin and simvastatin were selected, for several reasons: a) they are the most widely used lipid-lowering drugs in Spain (between 91 and 94% of the market share among statins with generic presentations available in the years of abstraction) [25], b) they are available in brand-name and generic presentations over a long period of time, which makes them suitable for prolonged follow-up, c) both statins are indicated to lower high blood cholesterol levels [6], d) the variability in outcomes is reduced considerably on analyzing only two statins, and e) there is little evidence regarding the relationship between these variables in real life, in either the international literature or in our healthcare setting, so this research may be of interest.

### Outcomes

#### Adherence

The adherence rate was defined according to the criteria of the International Society for Pharmacoeconomics and Outcomes Research (ISPOR) and calculated based on use/medication possession ratio (MPR) and treatment persistence [26]. *MPR* was assessed from the first to the last prescription and represents the number of days of medication supplied divided by the number of treatment days (from the index date). *Persistence* was defined as the time, measured in days, without stopping the initial treatment or switching to another medication at least 30 days after the initial prescription. It is expressed as the difference between the date of first supply (enrollment) and the date of last supply, plus the number of days that would be covered by the last supply (30 days).

#### Resource utilization and cost analysis

The societal and the Spanish NHS perspectives were taken into account to calculate healthcare and indirect costs. Healthcare costs (direct costs) were considered to be those relating to healthcare activity (medical visits, days of hospitalization, emergency visits, diagnostic and therapeutic requests, etc.) performed by healthcare professionals. Non-healthcare costs (indirect costs) were considered to be those relating to work productivity loss (days of sick leave due to temporary disability). Cost was expressed as mean cost per patient (average per unit) throughout the analysis period. Table 1 shows unitary costs of healthcare resources and days of sick leave applied in the economic valuations (in € for year 2016). Prices were based on the sites’ analytical accounting, except medication and days of sick leave. Medications were quantified by retail price per pack at the time of dispensing from the Community Pharmacy (according to the Drug Catalogue of the General Council of Associations of Official Pharmacists of Spain. Available from: https://botplusweb.portalfarma.com/). For this research, beside statins which both brand-name and generic have the same reference price to be funded by the NHS, the cost of medication included all drugs related with treatment of any possible cardiovascular risk factor and were quantified at their recommended retail price as this is the price funded by the NHS normally.

**Table 1:** Breakdown of costs per unit and work productivity losses (2016)

Healthcare and non-healthcare resources	Unit costs (€)
Medical visits	
Primary care medical visit	23.19
Emergency medical visit	117.53
Hospitalization (one day)	320.90
Specialized care medical visit^a^	92.00
Complementary tests	
Laboratory tests	22.30
Conventional radiology	18.50
Diagnostic/therapeutic tests^b^	37.12
Drug prescription	RRP_VAT_
Work productivity — Indirect costs	
Cost per day not worked	101.21

Source of healthcare resources: analytical accounting done by the authors and the INE. RRP: recommended retail price. Values are expressed in euros

^a^Only in respiratory medicine, cardiology, endocrinology and internal medicine departments

^b^Related to plasma lipid assessment

Days of occupational disability and productivity losses were quantified according to the average inter-professional wage (source: Spanish Statistical Office [*Instituto Nacional de Estadística*, INE]) [27]. The analysis did not take into account non-healthcare direct costs, i.e., “out-of-pocket” costs or costs paid by the patient/family, as these are not recorded in the database and patients themselves could not be accessed through the retrospective collection of existing records.

### Clinical effectiveness

The following clinical chemistry parameters obtained at the start and end of the follow-up were considered: total cholesterol, serum triglycerides, high-density lipoprotein cholesterol (HDL-c) and low-density lipoprotein cholesterol (LDL-c) in mg/dL. As an approximation to the clinical effectiveness, the following were considered: 1) The achievement of therapeutic goals, which was evaluated based on the patient’s LDL-c value (reduction in mean and percentage). The therapeutic goal was considered to be achieved for LDL-c levels ≤2.59 mmol/L (100 mg/dL) or 1.81 mmol/L (70 mg/dL), depending on whether a cardiovascular event would already have occurred or not, respectively [3]. The measurement was taken at the start (index date) and end of the follow-up period (value closest to the date), 2) Incidence of a cardiovascular event during the 5-year follow-up, differentiating: a) ischemic heart disease (angina, acute myocardial infarction), and b) cerebrovascular accident (transient ischemic attack, stroke); and 3) death of the patient (all-cause mortality). Both the incidence of a cardiovascular event and all-cause mortality were expressed in the form of incidence (density) rate, and calculated as the ratio between the number of new cases that occurred during the follow-up period and the sum of the risk periods of each of the individual patients throughout the period specified. This rate was measured in observed cases per 1000 person-years.

### Statistical analysis

Basic descriptive statistics, such as means and proportions not requiring statistical comparisons, are presented in tables by group of interest in the analysis. Analyses requiring statistical comparisons were performed using SPSS Version 17 (SPSS Inc., Chicago, USA). The normality of the distribution was verified using the Kolmogorov–Smirnov test. Standard parametric and non-parametric univariate statistical tests suitable for both the data type and the comparison group were performed. In making comparisons across patient subgroups within the RedISS sample, maximum likelihood/regression models were applied to isolate the influence of each possible explanatory variable on the outcome parameter of interest. These included generalized linear models [28, 29]. To fulfill the initial analysis plan according to the objective, records from simvastatin and atorvastatin were separated into two groups depending on whether they included a brand-name or a generic statin. Before this, homogeneity of records for each statin, comparing brand-name versus generic was carried out to identify possible sources of heterogeneity within the same drug. Standard univariate analysis was carried out (see Additional file 1: Tables S1 and S2).

The Mantel-Haenszel Chi-square test was applied to calculate *p* values in comparisons of unadjusted incidence rates for cardiovascular events and all-cause mortality between generic and brand-name statins. Multivariate analyses adjusted for covariates were applied to compare primary endpoints between brand-name and generic statins. Binary multivariate logistic regression was used to calculate the odds ratio (OR) adjusted by age, sex, number of comorbidities, Charlson index, resource utilization band (RUB), proportion of subjects reaching their LDL-c goal at the start of therapy and statin type of the difference in the percentage of patients reaching their LDL-c goals at discontinuation. A multivariate Cox proportional regression model was applied to calculate cumulative probability of persistence with initial statin therapy during the 5-year follow-up period in the whole sample, in patients without previous cardiovascular events, as well as with a previous cardiovascular event. The hazard ratios (HRs) were adjusted by age, sex, number of comorbidities, Charlson index, RUB, proportion of subjects reaching their LDL-c goal at the start of therapy and statin type. Adjusted HR relative to brand-name statin of the incidence of both cardiovascular events or all-cause mortality were also fitted using a Cox proportional risk model with covariates (age, sex, number of comorbidities, Charlson index, RUB, proportion of subjects reaching their LDL-c goal at the start of therapy, statin type and prior cardiovascular event. Differences in costs between brand-name and generic statins were compared using analysis of variance (ANOVA) for unadjusted costs and general linear models adjusted for age, sex, number of comorbidities, Charlson index, RUB, proportion of subjects reaching their LDL-c goal at the start of therapy, statin type, prior cardiovascular event and treatment duration. A 95% confidence interval (CI) of the differences was calculated with 1000 non-parametric bootstrap iterations.

### Reporting guidelines

Consolidated Health Economic Evaluation Reporting Standards (CHEERS) reporting guidelines were used to write this article [30].

## Results

**Fig. 1:**
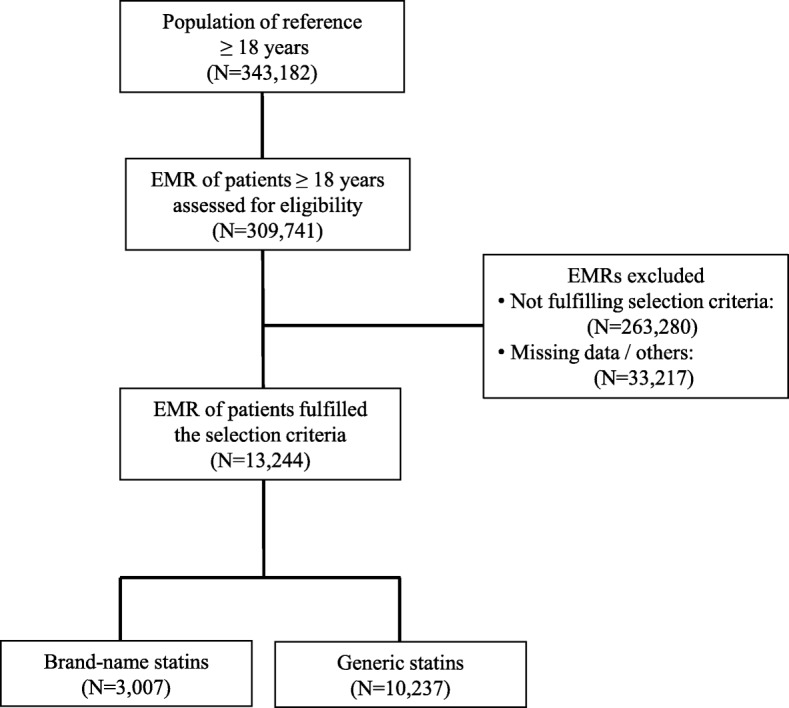
Flow diagram showing the selection procedure of patient electronic medical records for the investigational analysis. EMR = Electronic Medical Records. Population of reference and eligibility criteria are included in the text

From an initial screening, 343,182 EMRs of subjects 18 or more years of age were assigned to the sites and 13,244 records fulfilling enrollment criteria were recruited (Fig. 1). EMRs of patients receiving brand-name or generic statins were classified into 4 groups: brand-name atorvastatin (*N* = 1313; 20.9%), generic atorvastatin (*N* = 4957; 79.1%), brand-name simvastatin (*N* = 1694; 24.3%) and generic simvastatin (*N* = 5280; 75.7%), to test the homogeneity of brand-name versus generic within the same statin. Additional file 1: Tables S1 and S2 shows the baseline characteristics of patients analyzed according to the type of statin received before separating records into two groups: brand-name and generic statin, and the dosages used. Table 2 shows the demographic characteristics and comorbidities from the EMRs according to the use of generic or brand-name statins. Just over three-quarters of records (77.3%) corresponded to generic statins, with 52.7% corresponding to simvastatin. Mean age was 61.3 (standard deviation [SD]: 11.4) years and 52.6% were women. Significant differences at *p* < 0.05 level were not observed in the main characteristics according to type of statin, although presence of alcoholism, ischemic heart disease and cerebrovascular accident were at *p* < 0.1 level.

**Table 2:** Characteristics (demographics and comorbidity) at the start of therapy with generic or brand-name statin

Group	Brand-name	Generic	Total	p
Number of patients (%)	3007 (22.7%)	10,237 (77.3%)	13,244 (100%)	
Sociodemographic characteristics				
Average age (years)	61.4 (11.7)	61.2 (11.3)	61.3 (11.4)	0.501
Ranges:				
18–44 years	7.2%	7.7%	7.6%	
45–64 years	52.9%	51.9%	52.1%	
65–74 years	23.0%	25.9%	25.2%	
≥ 75 years	16.8%	14.6%	15.1%	0.234
Sex (female)	52.7%	52.5%	52.6%	0.827
Pensioners	50.2%	51.6%	50.5%	0.191
General comorbidity				
Average diagnoses	6.6 (3.6)	6.5 (3.3)	6.5 (3.3)	0.502
Charlson index	0.7 (1.0)	0.7 (1.0)	0.7 (1.0)	0.855
Average RUB	2.9 (0.7)	3.0 (0.7)	3.0 (0.7)	0.527
1 (very low comorbidity)	3.9%	3.4%	3.5%	
2 (low comorbidity)	15.4%	14.8%	14.9%	
3 (moderate comorbidity)	65.5%	66.7%	66.4%	
4 (high comorbidity)	13.2%	13.2%	13.2%	
5 (very high comorbidity)	2.1%	1.9%	1.9%	
Associated comorbidities				
Hypertension	50.2%	51.6%	50.5%	0.191
Diabetes mellitus	22.8%	23.0%	22.9%	0.786
Obesity	19.4%	19.8%	19.5%	0.658
Active smokers	24.0%	22.9%	23.7%	0.224
Alcoholism	3.5%	4.2%	3.7%	0.064
Ischemic heart disease	8.3%	9.4%	8.5%	0.057
Cerebrovascular accident	11.0%	9.9%	10.8%	0.091
Previous cardiovascular event	19.0%	18.3%	18.8%	0.382
Organ failure	17.3%	17.7%	17.4%	0.675
Dementia	2.4%	2.5%	2.5%	0.792
Depressive syndrome	21.5%	21.4%	21.5%	0.182
Malignant neoplasms	9.4%	9.0%	9.3%	0.537

Values expressed as percentage or mean (standard deviation), *p* statistical significance between brand-name vs. generic, *RUB* resource utilization band

**Fig. 2:**
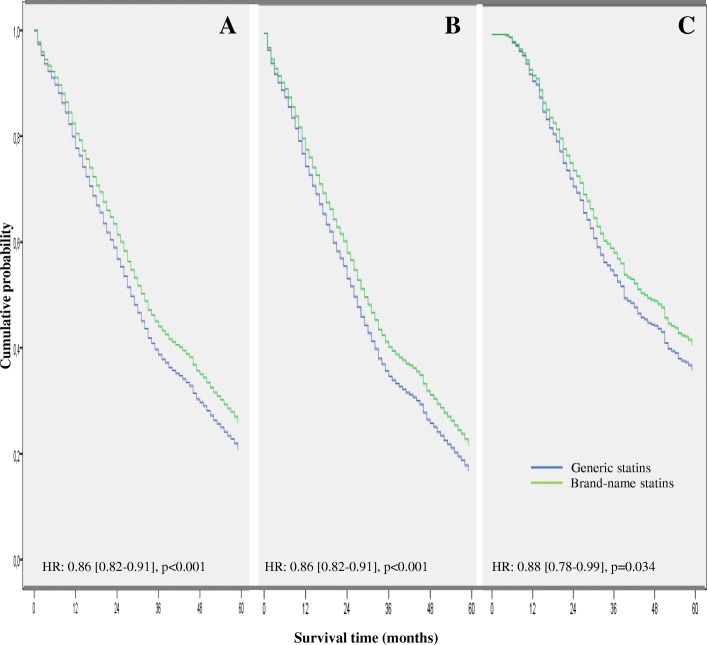
Cumulative probability of persistence with initial statin therapy during the 5-year follow-up period. Probability in the whole sample (graph **a**), patients without previous cardiovascular events (graph **b**) or with previous cardiovascular events (graph **c**). HR: Hazard Ratio with 95% confidence interval adjusted by age, sex, number of comorbidities, Charlson index, resource utilization band (RUB), proportion of subjects reaching their LDL-cholesterol goal at the start of therapy and statin type

Treatment persistence, MPR and doses administered are detailed in Table 3. Mean duration (SD) of generic statin treatment was significantly shorter than with brand-name statins: 32.0 (20.2) vs. 34.2 (20.5), *p* < 0.001), and the MPR was also significantly lower: 61.5% vs. 65.1%; *p* < 0.001. A significantly lower percentage of patients continued taking generic statins compared to brand-name statins at 60 months of follow-up (persistence): 20.7% vs. 25.9% with a hazard ratio (HR) 14% lower on average relative to the brand-name statin: 0.86 (95% CI: 0.82–0.91, *p* < 0.001). Treatment persistence was significantly lower from 12 months after the start of therapy: 77.7% vs. 80.3%, HR: 0.81 (0.74–0.89), *p* < 0.001 (Table 3, Fig. 2).

**Table 3:** Treatment persistence and medication possession ratio administered by group

Group	Brand-name	Generic	Total	p
Number of patients (%)	3007 (22.7%)	10, 237 (77.3%)	13,244 (100%)	
Time since diagnosis (months)	2.2 (2.4)	2.2 (2.3)	2.2 (2.4)	0.892
Median (P25–P75)	2.0 (1.0–3.0)	2.0 (1.0–3.0)	2.0 (1.0–3.0)	
Treatment possession (months)	22.3 (20.2)	19.6 (21.7)	20.2 (21.2)	< 0.001
Median (P25–P75)	21.0 (12.0–44.0)	19.0 (10.0–38.0)	20.0 (10.0–42.0)	
Treatment duration (months)	34.2 (20.5)	32.0 (20.2)	32.5 (20.3)	< 0.001
Median (P25–P75)	31.0 (15.0–60.0)	29.0 (14.0–55.0)	29.0 (14.0–56.0)	
Medication Possession Ratio				
Average	65.1%	61.5%	62.3%	< 0.001
95% CI	63.8–66.2%	60.1–62.2%	61.8–62.9%	
Percentage of patients on treatment at different cut-off point				Treatment persistence (HR [95% CI])^a^
12 months	80.3%	77.7%	78.3%	0.81 [0.74–0.89], *p* < 0.001
24 months	60.9%	56.9%	57.8%	0.93 [0.87–0.99], *p* = 0.021
60 months	25.9%	20.7%	21.9%	0.86 [0.82–0.91], *p* < 0.001

Values expressed as percentage or mean (*SD* standard deviation), *p* brand-name vs. generic, *CI* confidence interval, *P25* 25th percentile, *P75* 75th percentile

^a^*HR* adjusted hazard ratio relative to brand-name statin (adjusted using a Cox proportional risk model with covariates (age, sex, number of comorbidities, Charlson index, resource utilization band, proportion of subjects reaching their LDL-cholesterol goal at the start of therapy, statin type and prior cardiovascular event); *P* percentile

**Fig. 3:**
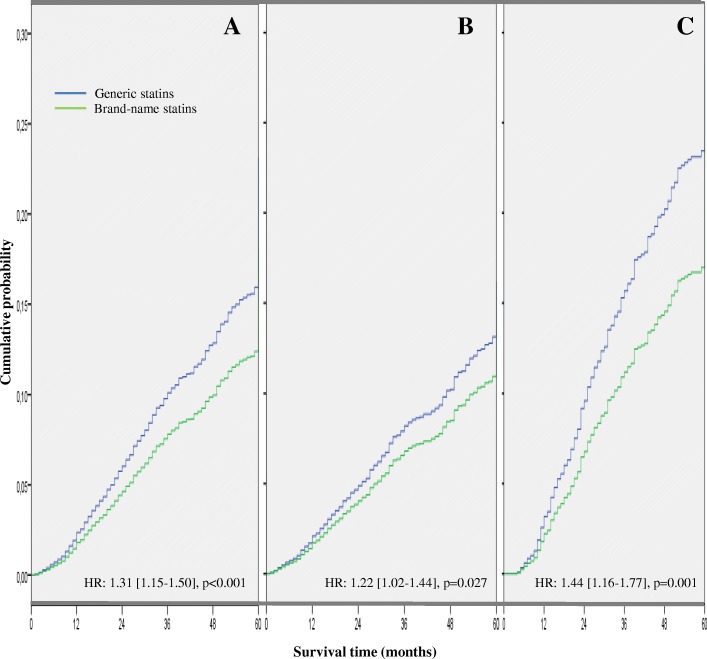
Cumulative probability of cardiovascular event with initial statin therapy during the 5-year follow-up period. Probability in the whole sample (graph **a**), patients without previous cardiovascular events (graph **b**) or with previous cardiovascular events (graph **c**). HR: Hazard Ratio with 95% confidence interval adjusted by age, sex, number of comorbidities, Charlson index, resource utilization band (RUB), proportion of subjects reaching their LDL-cholesterol goal at the start of therapy and statin type

**Fig. 4:**
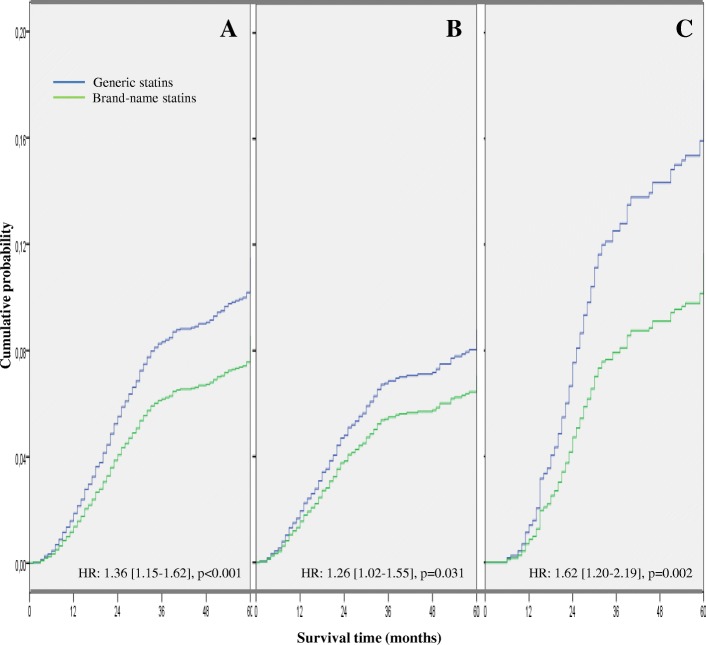
Cumulative probability of all-cause mortality with initial statin therapy during the 5-year follow-up period. Probability in the whole sample (graph **a**), patients without previous cardiovascular events (graph **b**) or with previous cardiovascular events (graph **c**). HR: Hazard Ratio with 95% confidence interval adjusted by age, sex, number of comorbidities, Charlson index, resource utilization band (RUB), proportion of subjects reaching their LDL-cholesterol goal at the start of therapy and statin type

The distribution of the different clinical chemistry parameters, therapeutic goals, incidence of cardiovascular events and all-cause mortality according to group analyzed are detailed in Table 4. While no significant differences were observed in the lipid parameters or in the percentage of patients who met their therapeutic goals at the start of the statin treatments, the probability of reaching their therapeutic goals during the 60-month follow-up period was on average 13% lower with the generic compared to with the brand-name statins; OR: 0.87 (0.80–0.95), *p* = 0.003; as a result, 39.2% of patients met their therapeutic goals with generic vs. 42.0% with brand-name statins. These findings were due to the significantly lower reduction in LDL-c levels with generic compared to brand-name statins: − 13.6 mg/dL vs. -17.0 mg/dL, respectively; *p* < 0.001 (Table 4). The crude incidence rate of cardiovascular events in number of cases per 1000 person-years was significantly higher in those who received therapy with generics compared to those with brand-name statins: 39.56 (37.24–41.99) vs. 31.48 (27.85–35.46), *p* < 0.001, with a 31% higher adjusted hazard ratio for having an event, HR: 1.31 (1.15–1.50), *p* < 0.001 (Fig. 3). These results were observed for both stroke and coronary events (Table 4). Likewise, the crude rate of all-cause mortality was significantly higher in those who received generic statins: 25.09 (23.25–27.04) vs. 18.82 (16.03–21.95). *p* < 0.001, with a 36% higher adjusted hazard ratio for death, HR: 1.36 (1.15–1.62), *p* < 0.001 (Fig. 4).

**Table 4:** Patient Outcomes: lipid panel variations, patients who have reached their lipid goals and incidence rate of cardiovascular events and all-cause mortality

Group	Brand-name	Generic	Total	p
Number of patients (%)	3007 (22.7%)	10,237 (77.3%)	13,244 (100%)	
Clinical chemistry parameters				
Total cholesterol, mg/dL; initial	223.9 [222.7–225.1]	223.2 [222.4–224.0]	223.3 [222.8–223.8]	0.489
Total cholesterol, mg/dL; final	189.4 [188.1–190.7]	192.8 [191.9–193.7]	192.0 [191.4–192.6]	< 0.001
Difference (initial – final)	−34.5	−30.4	−31.3	< 0.001
Triglycerides, mg/dL; initial	141.7 [140.2–143.2]	142.5 [141.6–143.4]	142.3 [141.7–142.9]	0.619
Triglycerides, mg/dL: final	129.8 [128.5–131.1]	133.8 [133.1–134.5]	132.9 [132.4–133.4]	0.014
Difference (initial – final)	−11.9	−8.7	−9.4	< 0.001
High-density lipoprotein cholesterol, mg/dL; initial	50.1 [48.3–51.9]	50.2 [49.0–51.4]	50.2 [49.3–51.1]	0.993
High-density lipoprotein cholesterol, mg/dL; final	55.7 [53.8–57.6]	54.3 [53.0–55.6]	54.5 [53.6–55.4]	< 0.001
Difference (initial – final)	5.6	4.1	4.3	0.035
Low-density lipoprotein cholesterol, mg/dL; initial	135.6 [133.7–137.5]	135.1 [133.6–136.6]	135.2 [134.3–136.1]	0.485
Low-density lipoprotein cholesterol, mg/dL; final	118.6 [116.8–120.4]	121.5 [120.1–123.0]	120.8 [120.2–121.4]	< 0.001
Difference (initial – final)	−17.0	−13.6	−14.4	< 0.001
Patients who have reached LDL-c goal (%)*				
At the start of statin therapy	17.9 [16.5–19.3]	17.7 [16.9–18.4]	17.7 [17.1–18.4]	0.811
At discontinuation	42.0 [40.2–43.7]	39.2 [38.3–40.2]	39.9 [39.0–40.7]	OR; 0.87 [0.80–0.95], *p* = 0.003
Absolute variation	24.1	21.5	22.2	< 0.001
Patient Outcomes (IR)**				
- Any cardiovascular event	31.48 [27.85–35.46]	39.56 [37.24–41.99]	37.63 [35.65–39.69]	< 0.001
­ CHD event	14.41 [11.98–17.18]	18.76 [17.17–20.45]	17.71 [16.36–19.14]	0.008
­ Stroke	17.08 [14.43–20.07]	20.81 [19.13–22.59]	19.91 [18.48–21.43]	0.033
- All-cause mortality	18.82 [16.03–21.95]	25.09 [23.25–27.04]	23.59 [22.03–25.23]	< 0.001

Values expressed as mean with standard deviation or 95% confidence interval in brackets, CHD: Coronary Heart Disease; IR: unadjusted cumulative incidence rate in number of cases per 1000 person-years, *OR: Odds ratio, adjusted by age, sex, number of comorbidities, Charlson index, resource utilization band (RUB), proportion of subjects reaching their LDL-cholesterol (LDL-c) goal at the start of therapy, statin type and prior cardiovascular event; **p values using the Mantel-Haenszel Chi-square test

Table 5 shows the comparison of brand-name vs. generic statins in resource use and costs. Of the total costs, 70.4% corresponded to healthcare costs (direct) and 29.6% to non-healthcare costs (productivity losses due to sick leave). Subjects undergoing treatment with brand-name statins used fewer healthcare resources, specifically in primary care visits (48.1 vs. 56.4; *p* < 0.001), specialized care visits (8.3 vs. 10.0; *p* < 0.001) and hospital emergencies (7.0 vs. 8.3; *p* < 0.001). The average per unit of the annual total cost (95% CI) of subjects undergoing treatment with brand-name vs. generic statins corrected for covariates was lower: €11,574 (11,103-12,045) vs. €12,978 (12,762-13,194), *p* = 0.001; difference: -€1404 (885–1922) in 60 months of follow-up. These differences were mainly due to lower healthcare costs: €7980 (7853-8808) vs. €9118, (9059-9176), *p* < 0.001; the work productivity losses, while lower, did not reach statistical significance.

**Table 5:** Resource utilization and associated costs (€) by group

Group	Brand-name	Generic	Total	P
Number of patients (%)	3007 (22.7%)	10,237 (77.3%)	13,244 (100%)	
Resource utilization				
Medical visits (primary care)	48.1 (27.0)	56.4 (26.4)	54.5 (26.8)	< 0.001
Laboratory tests	14.3 (9.7)	17.1 (10.3)	16.4 (10.2	< 0.001
Conventional radiology	8.0 (7.6)	10.7 (7.7)	10.1 (7.7)	< 0.001
Complementary tests	11.4 (4.4)	15.2 (4.2)	14.3 (4.5)	< 0.001
Days of hospitalization	1.1 (3.8)	1.8 (5.6)	1.6 (5.2)	< 0.001
Medical visits (hospital)	8.3 (7.4)	10.0 (7.9)	9.6 (7.8)	< 0.001
Emergency room visits (hospital)	7.0 (9.0)	8.3 (9.8)	8.0 (9.7)	< 0.001
Days of occupational disability	36.6 (101.1)	37.6 (105.0)	37.4 (104.1)	0.636
Unadjusted costs				
Healthcare costs	8246 (3699)	9071 (4093)	8927 (4039)	< 0.001
Costs in primary care	6320 (2658)	6605 (2657)	6555 (2659)	< 0.001
Medical visits	1105 (630)	1307 (613)	1272 (620)	< 0.001
Laboratory tests	315 (213)	380 (229)	369 (228)	< 0.001
Conventional radiology	149 (142)	199 (142)	190 (143)	< 0.001
Complementary tests	438 (169)	578 (158)	553 (169)	< 0.001
Drugs^a^	4314 (2377)	4141 (2359)	4171 (2363)	0.002
Costs in specialized care	1927 (2136)	2466 (2640)	2372 (2567)	< 0.001
Days of hospitalization	332 (1180)	568 (1783)	527 (1696)	< 0.001
Medical visits	763 (691)	923 (725)	895 (721)	< 0.001
Emergency room visits	832 (1098)	976 (1148)	951 (1141)	< 0.001
Non-healthcare costs (productivity loss)	3562 (10076)	3806 (10631)	3764 (10536)	0.327
Total costs (€)	11,808 (10854)	12,877 (11969)	12,691 (11789)	< 0.001
*Adjusted costs*^*b*^(€)	Brand-name	Generic	Difference	p
Healthcare costs	7980 (7853–8808)	9118 (9059–9176)	1137 (997–1277)	< 0.001
Primary care healthcare costs	6070 (6024–6116)	6653 (6632–6674)	583 (532–633)	< 0.001
­ Medication cost	4110 (4041–4178)	4188 (4156–4219)	78 (11–149)	0.028
Specialized healthcare costs	1910 (1805–2016)	2465 (2417–2514)	555 (439–671)	< 0.001
Non-healthcare costs (productivity loss)	3594 (3161–4026)	3860 (3662–4058)	267 (101–843)	0.272
Total costs	11,574 (11103–12,045)	12,978 (12762–13,194)	1404 (885–1922)	0.001

Values expressed as mean (*SD* standard deviation), *p* brand-name vs. generic, *95% CI* 95% confidence interval in parenthesis

^a^Drugs include statin costs plus all other possible treatments

^b^Adjusted by age, sex, number of comorbidities, Charlson index, resource utilization band, proportion of subjects reaching their LDL-cholesterol goal at the start of therapy, statin type, prior cardiovascular event and treatment duration. 95% confidence intervals of the differences calculated with non-parametric 1000 bootstrap iterations

### Analysis according to previous occurrence of a cardiovascular event

The lower probability of persistence at 60 months with generic statins was observed both in the overall sample and when this was divided into two subgroups according to previous occurrence of a cardiovascular event (Fig. 2); the likelihood of continuing to receive a statin was, respectively, 14% or 12% lower on average for the generic statin in patients without and with a previous cardiovascular event (*p* < 0.05 in both cases). Table 6 shows the main variables according to the absence/presence of previous cardiovascular events. In the absence of previous CVD, patients using generic vs. brand-name statins showed lower achievement of the therapeutic goals: 37.2% vs. 39.46%, OR: 0.89 (0.80–0.99), *p* = 0.025. Similar results were observed in patients with a previous cardiovascular event: 48.0% vs. 53.56%, OR: 0.78 (0.64–0.95), *p* = 0.015. The crude incidence rate of cardiovascular events in number of cases per 1000 person-years was higher in those who received therapy with generics than with brand-name statins (Table 6), particularly in subjects with a previous history of a cardiovascular event, with an adjusted hazard ratio for having an event between 22 and 44% higher according to whether they had or did not have a previous event, respectively, HR: 1.22 (1.02–1.44)], *p* = 0.027 and HR: 1.44 (1.16–1.77), *p* = 0.001 (Fig. 3). Similarly, the crude rate of all-cause mortality was higher in those who received generic statins, especially in patients with previous CVD, with an adjusted hazard ratio for death between 26 and 62% higher in patients receiving generic statins: HR: 1.26 (1.02–1.55), *p* = 0.031 and HR: 1.62 [1.20–2.19], *p* = 0.002, respectively (Fig. 4).

**Table 6:** Main patient outcomes according to previous major cardiovascular event

Group	No previous cardiovascular event			Previous cardiovascular event		
	Generic	Brand-name	p	Generic	Brand-name	p
Number of patients, %	*N* = 8292 (77.1%)	*N* = 2457 (22.9%)		*N* = 1280 (78.0%)	*N* = 550 (22.0%)	
Sociodemographics						
Age, mean (SD), years	59.9 (11.3)	60.1 (11.6)	0.427	67.0 (9.6)	67.2 (10.3)	0.640
Sex (female)	54.5%	54.9%	0.681	44.1%	42.9%	0.630
Comorbidity						
Number of comorbidities	6.3 (3.2)	6.4 (3.5)	0.198	7.5 (3.4)	7.8 (4)	0.087
Charlson index	0.6 (1.0)	0.6 (0.9)	0.675	1.2 (1.0)	1.3 (1.1)	0.935
RUB	2.9 (0.7)	2.9 (0.7)	0.485	3.3 (0.8)	3.3 (0.8)	0.755
Adherence						
Medication possession ratio (%)	59.4%	62.6%	*p* = 0.015	71.0%	76.0%	*p* = 0.002
Treatment persistence (%, HR)*	17.0%	22.8%	0.86 [0.82–0.91]; *p* < 0.001	36.1%	42.0%	0.88 [0.78–0.99]; *p* = 0.034
Patients who have reached LDL-c goal (%)**						
At the start of statin therapy	18.5 [17.7–19.3]	18.8 [17.3–20.3]	0.710	14.1 [12.2–16.0]	13.6 [10.8–16.5]	0.764
At discontinuation	37.2 [36.2–38.2]	39.4 [37.5–41.3]	OR; 0.89 [0.80–0.99]; *p* = 0.025	48.0 [45.2–50.7]	53.5 [49.3–57.6]	OR; 0.78 [0.64–0.95]; *p* = 0.015
Absolute variation	18.7	20.6	0.035	33.8	39.8	0.014
Patient Outcomes (IR)***						
- Cardiovascular event	29.08 [26.82–31.50]	24.69 [21.06–28.77]	0.062	74.42 [67.89–81.42]	55.41 [45.41–66.96]	0.006
- All-cause mortality	20.51 [18.61–22.55]	16.86 [13.88–20.29]	0.064	39.71 [34.98–44.91]	26.41 [19.67–34.73]	0.007
Costs (€)****	Generic	Brand-name	Difference	Generic	Brand-name	Difference
Healthcare costs	8365 [8285–8445]	7751 [7584–7918]	614 [432–796]; *p* < 0.001	12,081 [11890–12,271]	10,421 [10078–10,763]	1660 [1280–2040]; *p* < 0.001
Productivity losses	1101 [1047–1156]	981 [873–1089]	120 [12–232]; *p* = 0.068	15,337 [14443–16,230]	14,890 [13007–16,773]	447 [117–777]; *p* = 0.683
Total costs	9466 [9368–9565]	8732 [8536–8928]	734 [516–952]; *p* = 0.001	27,417 [26523–28,311]	25,310 [23512–27,110]	2107 [1805–2409]; *p* < 0.001

Values expressed as mean with standard deviation or 95% confidence interval (CI) in brackets; IR: unadjusted cumulative incidence rate in number of cases per 1000 person-years, ^*^HR: adjusted hazard ratio relative to brand-name statin (adjusted using a Cox proportional risk model with covariates (age, sex, number of comorbidities, Charlson index, resource utilization band [RUB], proportion of subjects reaching their LDL-cholesterol (LDL-c) goal at start of therapy, statin type and prior cardiovascular event); ^**^OR: Odds ratio, adjusted by age, sex, number of comorbidities, Charlson index, RUB, proportion of subjects reaching their LDL-c goal at the start of therapy and statin type; ^***^p values using the Mantel-Haenszel Chi-square test.^****^Adjusted by age, sex, number of comorbidities, Charlson index, RUB, proportion of subjects reaching their LDL-c goal at the start of therapy, statin type, prior cardiovascular event and treatment duration. 95% CI calculated with 1000 non-parametric bootstrap iterations

Table 6 also shows the comparison of brand-name vs. generic statins in resource use and costs. The average per unit of the annual total cost (95% CI) of subjects undergoing treatment with brand-name vs. generic statins corrected for covariates was significantly lower, both in the absence and presence of previous CVD; adjusted differences of 734 [516–952], *p* = 0.001 and 2107 [1805-2409]; *p* < 0.001, respectively, at 60 months of follow-up. These differences were also due to lower healthcare costs.

## Discussion

A generic drug is known to have the same qualitative and quantitative composition in terms of active substance and pharmaceutical form as the reference (brand-name) medicinal product, with proven bioequivalence (bioavailability) [13]. Nevertheless, generic versus brand-name drugs may differ in terms of excipient composition and outer appearance, which may result in problems of bio-appearance (type of packaging, tablet form, etc.), particularly for aging patients who are taken several medicines concomitantly [19, 24]. In Spain, the entry into the market of these drugs has helped to reduce pharmaceutical expenditure for the Spanish NHS, although, at present, both generic and brand-name drugs have the same acquisition cost as there is a reference price system for funded medicines [13]. In view of this, there should be no pharmacological arguments that indiscriminately prevent the prescription of brand-name or generic drugs.

The findings observed in this retrospective investigational research reveal that patients who started treatment with a generic statin compared to the brand-name drug were associated with lower treatment adherence, in terms of both percentage of days with medication possession (MPR ratio) and also days of persistence, which translated into poorer clinical outcomes (reduction in LDL-c levels, incidence of CVE), resulting in higher use of resources and sizeable healthcare costs for the Spanish NHS. The large sample size obtained, a long 5-year follow-up period, and consistency of outcomes with the two molecules studied should be considered strength of the investigation. It should be noted that there are few observational studies in real-world conditions in the literature consulted which, while it makes it difficult to compare results, highlights the fact that this investigation is unique. Thus, these findings might be considered of clinical and economic relevance. Persistence and adherence over time during the 5-year follow-up period were poor both for generics and for brand-name as well, although significantly worst for generic drugs. In this respect, abundant evidence shows that between 25 and 50% of patients fail to comply with treatment in the first 2 years of therapy [6–8]. Our figures are similar to or perhaps slightly higher than those reported (though still low). There might be several explanations for this: a) our method of measuring persistence, b) the dose indicated by the physician when initiating the treatment, c) ours is a more recent study, d) these are patients who require care (regularly attend check-ups), and/or e) they are subject to specific follow-up nursing care. Nonetheless, our results are consistent with other published findings [9, 10, 26]. In this respect, as well as known reasons for non-adherence, which may be intentional (sociodemographic factors, side effects, lack of understanding of treatment or health status, etc.) or unintentional (failure to remember how to take the medication correctly, etc.), the results of the investigation show that administration of a generic drug could be considered an additional factor to be taken into account [17, 19, 26, 31]. The appearance of the medicinal product (not measured in the investigation) might influence our results and affect the poorer adherence seen with generic drugs here. These factors include a different appearance (in terms of color and shape), a lack of certain presentations (delayed release or delayed absorption), variability in terms of excipients, a copayment effect or even a nocebo effect [31, 32].

The use of resources and costs were lower in patients treated with brand-name vs. generic drugs. The temporal relationship between lack of adherence and worst persistence, lower clinical effectiveness and greater use of healthcare resources is beyond doubt, and is consistent with the literature consulted [31–35]. Our research found such poorer persistence for the generic statin; approximately the probability of keeping taken the original therapy was a 14% lower with generics than with brand names, and this was regardless of the occurrence of a previous CVE [32, 33, 36]. Moreover, patients undergoing treatment with generic statins showed lower reduction in their therapeutic goals than those receiving a brand-name statin (approximately 13% chance). As a consequence, and after adjusting for confounding covariates, it should be noted that with generics the probability of suffering a CVE was higher on an average 31% during the 5-year follow-up (22 to 44% depending of previous occurrence of a CVE). Also, the adjusted probability of all-cause death was significantly higher with generics (on an average 36%), and this was irrespective of previous occurrence of a CVE; 26% in subjects with no previous CVE and 62% with previous CVE. In this aspect, Tran et al. [37] reported that the use of generic drugs is associated with a reduction in therapeutic goals of LDL-c in the treatment of dyslipidemia. Gagne et al. [38], in a prospective study, however found that patients who started treatment with brand-name versus generic statins had higher rates of non-adherence and cardiovascular episodes. While these data cannot be generalized, in fact Gagne et al. pointed out that such finding could be due to the ample differential acquisition costs of brand-name statins in comparison with generic in the US, these differences are consistent with other published studies [33]. On the other hand, not only poorer clinical outcomes were showed with generic drugs, but also it was observed a correlates with higher utilization of resources (all-type healthcare costs in particular, but not sick leaves), that was translated into significantly higher costs for the Spanish NHS; on average €1137 per patient (€614 in subjects without previous CVE and €1660 in patients with a previous CVE) in a 5-year follow-up period. This means and extra cost while the patient is on generic versus brand-name therapy of about €35.5 per patient per month in healthcare costs (of which €2.4 are drugs). As resources are limited and there are healthcare budget constraints, this sizeable cost should alert both clinicians and health decision makers of the Spanish NHS when facing hypercholesterolemia, as this condition is highly prevalent in the community [5].

Arguments in favor of and against generic drugs are not without controversy as commented above [14, 15]. By way of example, reviews conducted by Kesselheim et al. [39] and Manzoli et al. [40] defended the similar clinical efficacy between the brand-name and the generic drug. Mano et al. [41], in a retrospective study, reported that changing from brand-name (*N* = 147) to generic atorvastatin (*N* = 135) did not affect treatment persistence (85.9% vs. 73.5%) in patients, after 180 days of treatment. On the basis of 266 patients, Loch et al. [42] concluded that brand-name vs. generic atorvastatin achieved similar results (total and LDL cholesterol) in the clinical management of dyslipidemia, except for HDL-c levels (better with brand-name atorvastatin). Nevertheless, to our understanding, the following should be assessed: whether the sample size obtained was adequate (statistical power), where the balance would be between statistical significance and clinical relevance/impact, and when meta-analyses are performed based on these studies. Other authors, in contrast, concluded their publications with a number of recommendations. Candido et al. [43] suggest that the use of a generic drug may underestimate the effect of adherence to some medicinal products (single dose, delayed absorption); therefore, medicinal products administered to patients with chronic pain should be personalized to better meet analgesic needs and ensure patient safety. Fraeyman et al. [44], based on a survey of 1636 patients, recommended highlighting the name of the active substance on medication packaging labels to prevent health risks, especially among older patients. Colombo et al. [31] concluded that their results were consistent with studies supporting the possibility that a change in the package appearance each time a new generic drug prescription is dispensed may create confusion and reduce patient adherence, which may in turn influence clinical effectiveness and safety. Our results could support these contributions. Like efficacy and bioequivalence between the brand-name and the generic drug, the results of this investigation suggest that changes in the *appearance* of the drug may have repercussions for patient safety, especially in chronic diseases, older patients and/or poly-medicated patients. Reducing variability in the appearance (image of the drug or similar) among chemically identical medicines could help to discourage treatment discontinuation [31, 32].

The potential limitations of this investigation are those inherent to its retrospective nature, such as disease under-recording and potential variability between professionals and patients due to the investigation’s observational design, the system of measurement used for the main variables, and the potential existence of a classification bias. In this regard, any inaccuracy in diagnostic coding in the diagnosis of hypercholesterolemia, or the lack of a variable that could affect the final results (socioeconomic status of patients, changes in the drug doses prescribed, changes in form and presentation in the generics, etc.) should be considered as a limitation of the investigation. However, to our understanding, the main deficiencies of the investigation were as follows: a) the selection bias on the part of the treating physician when starting a brand-name or generic treatment, since this was not done randomly, as is typical in a real-world situation, and b) the external validity of the results (generalization), since the investigation was conducted in institutions that provide healthcare services, with similar organizational and clinical-management systems. Also, this investigation was not able to analyze possible side-effects of statins as these usually are not coded but including as clinical notes. Consequently, the results of the investigation should be interpreted with caution. Future efforts should focus on replicating this investigation at other healthcare institutions and on promoting intervention strategies intended to promote patient adherence to the treatments prescribed by their physicians.

## Conclusions

In conclusion, meaningful and significantly lower levels of treatment adherence and persistence were observed in patients with high LDL-cholesterol who first started therapy with generic in comparison to brand-name statins in routine medical practice in Spain. Also, compared with brand-name statin therapy, this retrospective cost-consequences analysis found that patients receiving generics were more unlikely to reach LDL-c goals, showed increased probability of having a major cardiovascular event and all-cause death at a higher cost to payers.

## Additional file

Additional file 1: **Table S1.** Sociodemographic and comorbidities at start of treatment comparing brand-name vs. generic by type of statin. **Table S2.** Distribution of statin dosages comparing brand-name vs. generic by type of statin. (DOCX 22 kb)

## Abbreviations

ANOVA: Analysis of variance
ATC: Anatomical Therapeutic Chemical
CHD: Coronary Heart Disease
CHEERS: Consolidated Health Economic Evaluation Reporting Standards
CI: Confidence interval
CVD: Cardiovascular disease
CVE: Cardiovascular event
EMR: Electronic medical records
HDL-c: High-density lipoprotein cholesterol
HR: Hazard ratio
ICD-9-CM: International Classification of Diseases (Ninth Revision), Clinical Modification
ICPC-2: International Classification of Primary Care version 2
INE: Instituto Nacional de Estadística (Spanish Statistical Office)
IR: Incidence rate
ISPOR: International Society for Pharmacoeconomics and Outcomes Research
LDL-c: Low density-lipoprotein cholesterol
MPR: Medication possession ratio
NHS: National Health System
OR: Odds ratio
P: percentile
RedISS: Red de Investigación en Servicios en Salud (Research net on Health Services)
RRP: Recommended retail price
RUB: Resource utilization band
SD: Standard deviation
SPSS: Statistical Package for Societal Sciences
USA: United States of America
