# Supplementary material for: Treatment persistence and adherence and their consequences on patient outcomes of generic versus brand-name statins routinely used to treat high cholesterol levels in Spain: a retrospective cost-consequences analysis
Source: Lipids Health Dis. 2018 Dec 6;17:277. doi: 10.1186/s12944-018-0918-y (PMC6284297; doi:10.1186/s12944-018-0918-y)
Supplement: Supplementary file 1 — Table S1. Sociodemographic and comorbidities at start of treatment comparing brand-name vs. generic by type of statin. Table S2. Distribution of statin dosages comparing brand-name vs. generic by type of statin. (DOCX 22 kb) [file 12944_2018_918_MOESM1_ESM.docx]

Table S1: Sociodemographic and comorbidities at start of treatment comparing brand-name vs. generic by type of statin.

| Type of statin | Atorvastatin | | | |  | Simvastatin | | | |
| --- | --- | --- | --- | --- | --- | --- | --- | --- | --- |
| Subgroup | Generic | Brand-name | Total | p |  | Generic | Brand-name | Total | p |
| Number of records, % | 4,957 (79.1%) | 1,313 (20.9%) | 6,270 (100%) |  |  | 5,280 (75.7%) | 1,694 (24.3%) | 6,974 (100%) |  |
| Mean age, years | 62.9 (10.5) | 62.9 (11.2) | 62.9 (10.6) | 0.801 |  | 59.7 (11.9) | 60.2 (12.0) | 59.8 (11,9) | 0.144 |
| Sex (women) | 51.5% | 53.2% | 51.8% | 0.279 |  | 53.4% | 52.4% | 53.2% | 0.461 |
| *General comorbidity* |  |  |  |  |  |  |  |  |  |
| Mean number of comorbidities | 6,8 (3,4) | 6,7 (3,5) | 6,8 (3,4) | 0,100 |  | 6,2 (3,1) | 6,6 (3,8) | 6,3 (3,3) | <0,001 |
| Charlson index | 0,8 (0,9) | 0,8 (1) | 0,8 (0,9) | 0,628 |  | 0,7 (1,1) | 0,7 (1,0) | 0,7 (1,1) | 0,465 |
| Mean resources utilization band | 3,0 (0,7) | 3,0 (0,7) | 3,0 (0,7) | 0,931 |  | 2,9 (0,7) | 2,9 (0,7) | 2,9 (0,7) | 0,607 |
| 1 (comorbilidad muy baja) | 2,6% | 3,7% | 2,9% |  |  | 4,2% | 4,0% | 4,2% |  |
| 2 (comorbilidad baja) | 13,6% | 11,9% | 13,2% |  |  | 16,0% | 18,1% | 16,5% |  |
| 3 (comorbilidad moderada) | 67,2% | 67,6% | 67,3% |  |  | 66,3% | 63,9% | 65,7% |  |
| 4 (comorbilidad elevada) | 14,6% | 14,5% | 14,6% |  |  | 11,8% | 12,1% | 11,9% |  |
| 5 (comorbilidad muy elevada) | 2,0% | 2,3% | 2,0% | 0,168 |  | 1,7% | 1,9% | 1,8% | 0,339 |
| *Comorbilidades asociadas* |  |  |  |  |  |  |  |  |  |
| Hipertensión arterial | 50,1% | 51,0% | 50,2% | 0,561 |  | 49,6% | 47,9% | 49,2% | 0,227 |
| Diabetes mellitus | 23,9% | 23,5% | 23,8% | 0,735 |  | 21,8% | 22,7% | 22,0% | 0,413 |
| Obesidad | 20,2% | 18,6% | 19,8% | 0,205 |  | 18,7% | 20,7% | 19,2% | 0,071 |
| Fumadores activos | 22,8% | 22,4% | 22,7% | 0,732 |  | 25,0% | 23,3% | 24,6% | 0,147 |
| Alcoholismo | 3,8% | 4,1% | 3,9% | 0,592 |  | 3,2% | 4,3% | 3,5% | 0,033 |
| Cardiopatía isquémica | 7,7% | 9,5% | 8,1% | 0,030 |  | 8,8% | 9,3% | 8,9% | 0,579 |
| Accidente vasculocerebral | 12,4% | 10,8% | 12,1% | 0,120 |  | 9,7% | 9,2% | 9,6% | 0,555 |
| ECV previo | 20,1% | 19,6% | 20,0% | 0,687 |  | 18,0% | 17,3% | 17,8% | 0,515 |
| Insuficiencias orgánicas | 18,9% | 18,5% | 18,8% | 0,757 |  | 15,9% | 17,0% | 16,1% | 0,271 |
| Demencias (todos los tipos) | 2,8% | 3,0% | 2,8% | 0,611 |  | 2,1% | 2,1% | 2,1% | 0,992 |
| Síndrome depresivo | 21,6% | 21,1% | 21,5% | 0,842 |  | 21,4% | 21,8% | 21,5% | 0,701 |
| Neoplasias malignas | 9,9% | 9,4% | 9,8% | 0,602 |  | 8,9% | 8,7% | 8,9% | 0,799 |

Valores expresados en porcentaje o media (desviación estándar), p: significación estadística. ECV: evento cardiovascular.

Table S2: Distribution of statin dosages comparing brand-name vs. generic by type of statin.

| Type of statin | Atorvastatin | | | | | | | |  | | Simvastatin | | | | | | | |
| --- | --- | --- | --- | --- | --- | --- | --- | --- | --- | --- | --- | --- | --- | --- | --- | --- | --- | --- |
| Subgroup | Generic | | Brand-name | | Total | | p | |  | | Generic | | Brand-name | | Total | | p | |
| Number of records, % | 4,957 (79.1%) | | 1,313 (20.9%) | | 6,270 (100%) | |  |  |  | | 5,280 (75.7%) | | 1,694 (24.3%) | | 6,974 (100%) | |  |  |
| 10 mg/day | | 35,5% | | 33,5% | | 35,1% | |  | | 18,1% | | 19,4% | | 18,4% | |  | |  |
| 20 mg/day | | 41,7% | | 42,4% | | 41,8% | |  | | 60,5% | | 60,3% | | 60,4% | |  | |  |
| 40 mg/day | | 13,3% | | 14,1% | | 13,4% | |  | | 21,4% | | 20,3% | | 21,2% | | 0,558 | |  |
| 80 mg/day | | 9,5% | | 10,0% | | 9,7% | | 0,691 | |  | |  | |  | |  | |  |
